# Comparison of Microbial Community Compositions of Injection and Production Well Samples in a Long-Term Water-Flooded Petroleum Reservoir

**DOI:** 10.1371/journal.pone.0023258

**Published:** 2011-08-15

**Authors:** Hong-Yan Ren, Xiao-Jun Zhang, Zhi-yong Song, Wieger Rupert, Guang-Jun Gao, Sheng-xue Guo, Li-Ping Zhao

**Affiliations:** 1 State Key Laboratory of Microbial Metabolism and School of Life Science & Biotechnology, Shanghai Jiao Tong University, Shanghai, China; 2 Institute of Oil Recovery Research, Shengli Oil Field Ltd., Dongying, China; Université Paris Sud, France

## Abstract

Water flooding plays an important role in recovering oil from depleted petroleum reservoirs. Exactly how the microbial communities of production wells are affected by microorganisms introduced with injected water has previously not been adequately studied. Using denaturing gradient gel electrophoresis (DGGE) approach and 16S rRNA gene clone library analysis, the comparison of microbial communities is carried out between one injection water and two production waters collected from a working block of the water-flooded Gudao petroleum reservoir located in the Yellow River Delta. DGGE fingerprints showed that the similarities of the bacterial communities between the injection water and production waters were lower than between the two production waters. It was also observed that the archaeal composition among these three samples showed no significant difference. Analysis of the 16S rRNA gene clone libraries showed that the dominant groups within the injection water were Betaproteobacteria, Gammaproteobacteria and Methanomicrobia, while the dominant groups in the production waters were Gammaproteobacteria and Methanobacteria. Only 2 out of 54 bacterial operational taxonomic units (OTUs) and 5 out of 17 archaeal OTUs in the injection water were detected in the production waters, indicating that most of the microorganisms introduced by the injection water may not survive to be detected in the production waters. Additionally, there were 55.6% and 82.6% unique OTUs in the two production waters respectively, suggesting that each production well has its specific microbial composition, despite both wells being flooded with the same injection water.

## Introduction

There is growing interest in the study of petroleum reservoir microbiota due to the prevalence of microbial enhanced oil recovery (MEOR) stimulated by increased global energy demand and depletion of oil reserves [1]. Many reports on microbial studies of petroleum reservoirs using culture-dependent and -independent methods have been published since Bastin *et al.* isolated sulfate-reducing bacteria (SRB) from production water in 1926 [2]. The typical groups, such as SRB, fermentative bacteria, iron-reducing bacteria and methanogenic bacteria, have been frequently reported in the microbial communities of oil reservoirs [3]. However, the continuous discovery of novel bacterial and archaeal phylotypes in oil reservoirs indicates the potential existence of undetected microbial assemblages in petroleum reservoirs [4]–[6].

To enhance oil recovery, a high proportion of petroleum reservoirs in the world have been extensively water flooded [7]. The injection water produced from oil-water separation of production waters is recycled into injection well through a semi-open system. The previous investigations of water flooded petroleum reservoirs suggest that they are complex ecosystems comprising a number of microorganisms [8]–[11]. The bacterial diversity of Huabei oil field, a continental high-temperature and water-flooded petroleum reservoir, was analyzed using clone library approach, and found 74 phylotypes with representative classes Gammaproteobacteria, Thermotogae, Epsilonproteobacteria, etc. [12]. At the same time, the archaeal community of the oil field was also characterized using clone library approach, and found 28 phylotypes composed of four orders of methanogens [13]. On the other hand, microorganisms in the water recycling system are injected back into the reservoirs during the flooding process [8], which possibly caused the change of the microbial community structure of petroleum reservoir. For this reason, investigating the microbial composition of injection water is important for understanding its effects on the ecosystem of petroleum reservoirs. Using DNA fingerprinting methods, such as denaturing gradient gel electrophoresis (DGGE) and terminal restriction fragment length polymorphism (T-RFLP) [14], [15], several studies have compared the microbial communities of injection water and production waters. These studies revealed that community structures of injection and production waters are different. However, due to the disadvantages of the fingerprinting approaches, it is difficult to compare the microbial communities of different samples in detail. How the structure of subsurface microbial community is affected by injected microorganisms in water-flooded oil reservoirs has rarely been studied thus far.

In this study, microbial communities of one injection water and two neighboring production waters from a hyperthermal, long-term water-flooded oil field were investigated using both polymerase chain reaction (PCR) fingerprinting and 16S rRNA gene clone library analysis. The results showed that most microorganisms in the injection water could not be detected in the production water, and each production well was composed of a unique microbial community.

## Results

### Physicochemical characteristics of the Gudao petroleum reservoir

The Gudao petroleum reservoir is located at Dongying in the Yellow River Delta of China, near the Bohai Sea. This oil field has been water flooded for over 30 years. The water content of production water in this reservoir is over 95%. The depths of the sampled petroleum horizons ranged from 1,173 m to 1,230 m, with a temperature of 69.5°C and a pressure of 12 MPa. The porosity of the reservoir was 33%, and air permeability was between 1.5–2.5 m^2^. The viscosity of the crude oil was 400–2,000 mPas. Three samples were used in this study: one injection water (W), one production water (C) abstracted from oil-bearing stratum Ng4^4^, and one production water (L) abstracted from oil-bearing strata Ng3^4^ and Ng3^5^. Physicochemical characteristics of the injection water and production waters are presented in Table 1.

**Table 1:** Physicochemical characters of the injection water and two production waters of the Gudao petroleum reservoir.

Parameters	Injection water samples	Production water samples	
	W (G1–6)	L (6–13)	C (3C15)
Temperature (°C)	40–50	69	69
pH	7.4–7.8	7.2–7.5	7.2–7.5
Depth (m)	/	1173–1230	1173–1230
Chemical characteristics (mg/l)			
Cl^−^	4045	3697	3183
CO_3_^2−^	33	/	/
HCO_3_^−^	854	1013	1113
Mg^2+^	41	/	41
K^+^ + Na^+^	2795	2661	2338
SO_4_^2−^	16	/	/
Ca^2+^	92	104	56
gas composition (%)			
CH_4_	Nd*	79.87	96.90
C_2_H_6_	Nd	6.95	0.99
C_3_H_8_	Nd	7.54	0.93
i-C_4_H_10_	Nd	1.40	0.15
N-C_4_H_10_	Nd	2.46	0.26
CO_2_	Nd	0.47	0.01

*not detected.

### PCR-DGGE analysis of the injection water and production water samples

**Figure 1:**
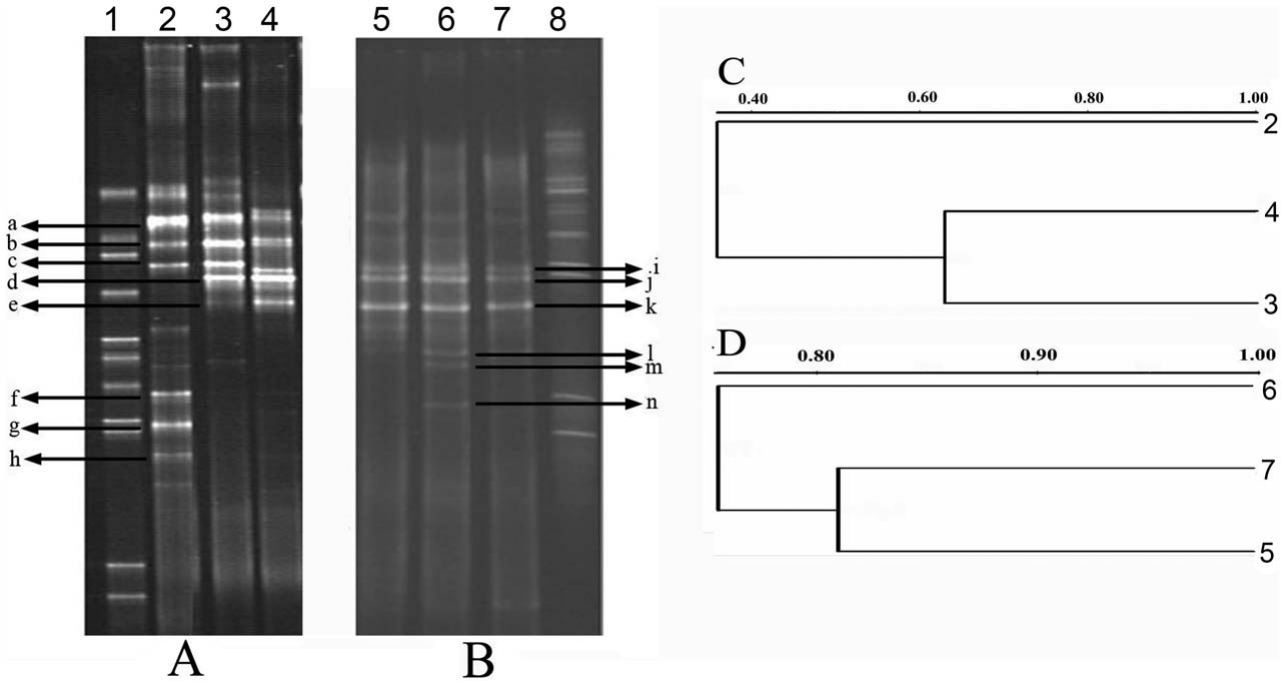
DGGE fingerprints of microbial communities of three samples. (A) Bacterial 16S rRNA gene V3 region PCR-DGGE profiles of injection and production waters. (B) Archaeal 16S rRNA gene V3 region PCR-DGGE profiles of injection water and production waters. (C) Clustering dendrogram of bacterial DGGE profiles. (D) Clustering dendrogram of archaeal DGGE profiles. 1, bacterial DGGE marker; 2 and 5, sample W; 3 and 7, sample C; 4 and 6, sample L; 8, archaeal DGGE marker.

DGGE analysis of a PCR-amplified V3 region of bacterial 16S rRNA genes showed that three dominant bands (a, b and c) were found in all three samples, and two dominant bands (d and e) were observed in C and L only. Several bands (f, g, h, etc.) were found in W but absent in C and L (Figure 1A). Statistic analysis (unweighted pair group method using arithmetic averages, UPGMA) revealed that the patterns of C and L shared 62.9% similarity. The W shared 29.1% similarity with L and 43.0% similarity with C (Figure 1C). The archaeal DGGE profiles showed that dominant bands (i, j and k) were observed in all three samples, while a few weak bands (l, m and n) were found only in L (Figure 1B). UPGMA analysis revealed that the patterns of these three samples shared at least 74% similarity (Figure 1D).

### Statistical analysis of the bacterial and archaeal 16S rRNA gene clone libraries

Three bacterial clone libraries (BW, BC and BL) were constructed using total genomic DNA. From these libraries, 360 bacterial clones were randomly selected and sequenced (Table 2). After discarding 34 chimeras, 326 sequences were divided into 66 bacterial OTUs. The coverage of the three clone libraries ranged from 86.5% to 96.9%. This result indicates that the 16S rRNA gene sequences represent the majority of the bacterial community of the samples in this study. The Shannon-Weaver and Simpson indexes showed that the diversity of bacteria in BW was higher than that in BC and BL. The low P values (<0.03) for UniFrac significance test between each pair of samples indicated that bacterial structures of these three samples had a marginally significant difference.

**Table 2:** Statistical analysis of bacterial and archaeal 16S rRNA gene clone libraries.

Library name	Bacterial library			Archaeal library		
	BC	BL	BW	AC	AL	AW
Number of raw sequences	71	69	220	33	35	98
Number of Chimeras	6	0	28	1	0	0
Number of analyzed sequences	65	69	192	32	35	98
Coverage (%)	96.9	91.3	86.5	90.6	82.9	91.9
OTU number	5	11	54	4	12	17
Shannon index (H)	0.92	1.34	3.14	0.41	2.07	1.77
Simpson (1-D)	0.49	0.58	0.90	0.18	0.83	0.65

Using the same analytical methods, three archaeal clone libraries (AW, AC and AL) were constructed, from which 166 clones were randomly selected and sequenced (Table 2). After discarding 1 chimera, 165 sequences were divided into 26 archaeal OTUs. The coverage of AL (82.9%) was lower than that of AC (90.6%) and AW (91.9%). The Shannon-Weaver and Simpson indexes showed that the diversity of archaea in AL was higher than that in AC and AW. Unifrac analysis showed marginally significantly different archaeal structures between three samples (P≤0.03).

### Phylogenetic analysis of microbial communities in samples

**Figure 2:**
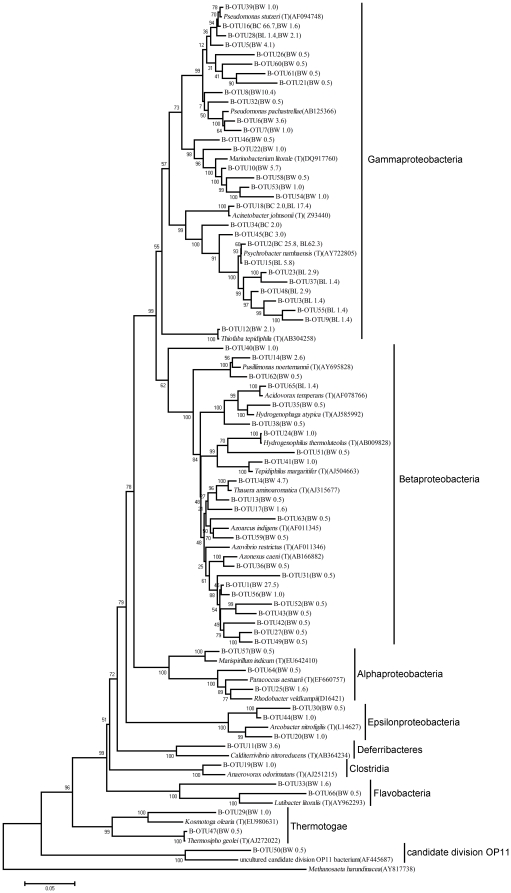
Phylogenetic neighbor-joining trees of representative OTUs in the bacterial clone libraries. Following the name of each OTU, the name of the sample and the percentage of this OTU was indicated in the parentheses. The numbers on the branches are bootstrap values obtained from 1000 bootstrap replicates. BW, sample W; BC, sample C; BL, sample L.

**Figure 3:**
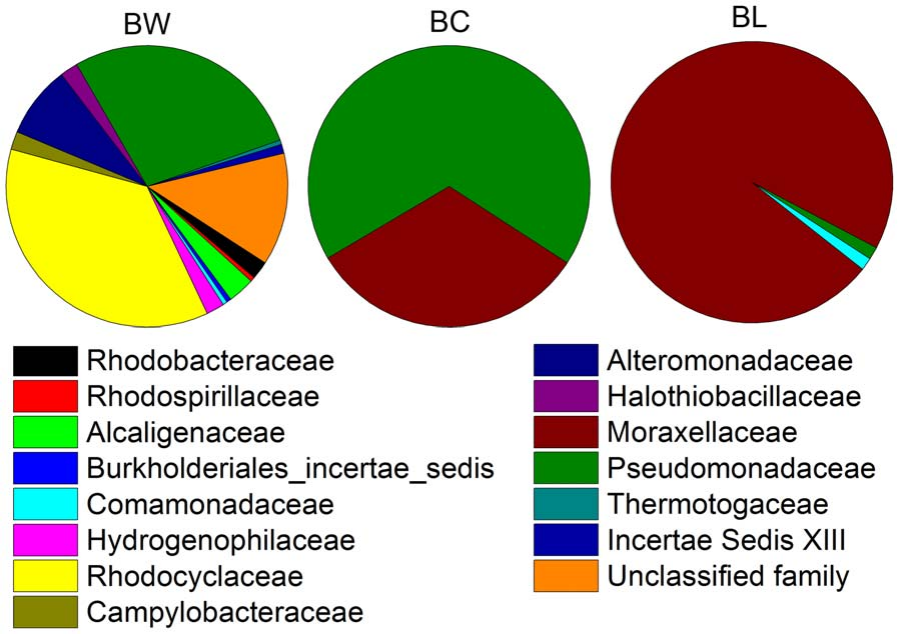
The bacterial composition of injection water and production waters at the family level. BW, sample W; BC, sample C; BL, sample L.

The compositional differences in bacterial communities were analyzed based on bacterial 16S rRNA gene sequences obtained from the three bacterial clone libraries (BW, BC and BL). In BW, 0.5% sequences were considered phylogenetically undefined bacteria, which shared less than 75% identity with reference sequences in the database. The remaining sequences were clustered within five phyla: Proteobacteria (91.2%), Deferribacteres (3.5%), Bacteroidetes (2.2%), Thermotogae (1.6%) and Firmicutes (1.0%) (Figure 2). At the family level, 13% of the total sequences could not be assigned to known families. The dominant families in BW were Rhodocyclaceae (36.5%), Pseudomonadaceae (28.1%) Alteromonadaceae (8.3%) and Alcaligenaceae (3.1%) (Figure 3). At the genus level, only 45.1% of all sequences were affiliated with specific genera. Of these genera, *Pseudomonas* (24.3%), *Thauera* (5.2%), *Marinobacterium* (6.7%) and *Pusillimonas* (3.1%) had its abundance higher than 3.0%. Unlike BW, except for one OTU (B-OTU65), the remaining bacterial sequences in BC and BL belonged to Gammaproteobacteria (Figure 2). In BC, all the sequences were affiliated with two families, Pseudomonadaceae (67.7%) and Moraxellaceae (32.3%). Among these families, 98.5% of sequences were affiliated with *Pseudomonas* (67.7%), *Psychrobacter* (29.3%) and *Acinetobacter* (1.5%). In BL, all of the sequences were assigned to three families, Moraxellaceae (97.1%), Pseudomonadaceae (1.5%) and Comamonadaceae (1.4%) (Figure 3). Among them, 94.1% of sequences were affiliated with four genera, *Psychrobacter* (73.9%), *Pseudomonas* (1.4%), *Acinetobacter* (17.4%) and *Acidovorax* (1.4%), respectively.

**Figure 4:**
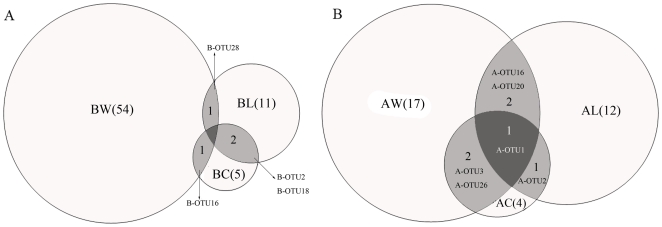
Venn diagram showing the distribution of OTUs. (A) Bacterial OTUs. (B) Archaeal OTUs. BW and AW, sample W; BC and AC, sample C; BL and AL, sample L. The number of OTUs in each sample is in parentheses. The number and name of OTUs shared by different samples are showed in the shaded part of the circles.

There were 66 bacterial OTUs (labeled as B-OTU) in these three samples (Figure 2 and Figure 4A). B-OTU16, related to *Pseudomonas stutzeri* (AF094748) (>98%), had an abundance of 1.6% in BW and 66.7% in BC but was not detected in BL. In previous literature, *P. stutzeri* has been shown to enhance crude oil biodegradation and increase rhamnolipid production [16]. B-OTU28, related to *Pseudomonas xanthomarina* (AB176954) (>98%), had an abundance of 2.1% in BW and 1.4% in BL but was not detected in BC. *P. xanthomarina* has been reported as a denitrifying bacterium isolated from marine ascidians [17]. Except the above two OTUs, 96.3% OTUs in BW were not detected in either of the two production waters. Two OTUs (B-OTU2 and B-OTU18) co-existed in BC and BL libraries, which were closely related to *Psychrobacter namhaensis* (AY722805) (>99%) and *Acinetobacter johnsonii* (HQ650820) (>98%), respectively. The proportion of B-OTU2 in BC and BL was 25.8% and 62.3%, and the proportion of B-OTU18 in BC and BL was 1.5% and 17.4%, respectively. However, 40.0% and 72.7% unique OTUs were found in BC and BL libraries, respectively. These results indicated that each production well appears to have a unique bacterial community despite water flooding with the same injection water for over 30 years.

**Figure 5:**
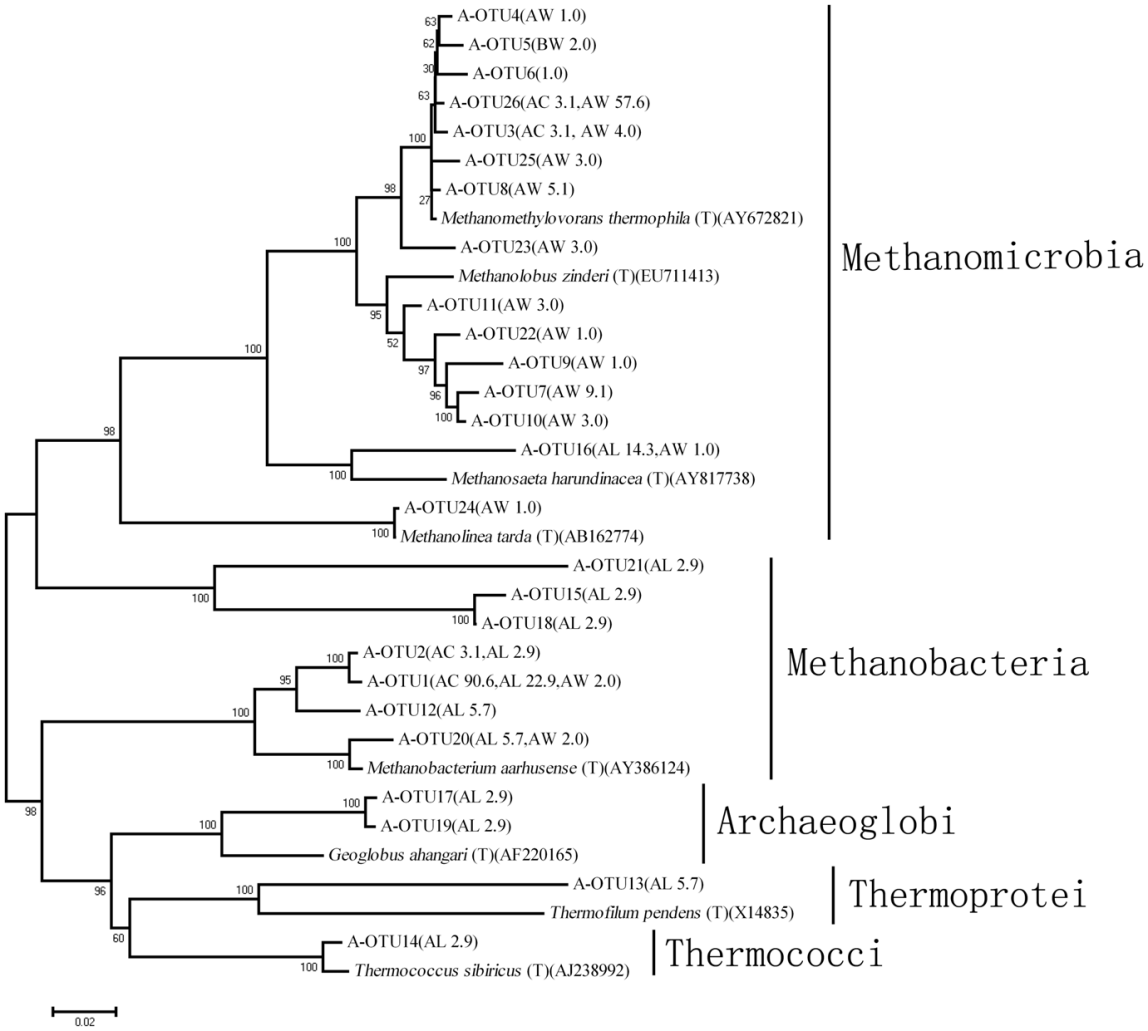
Phylogenetic neighbor-joining trees of representative OTUs in the archaeal clone libraries. Following the name of each OTU, the name of the sample and the percentage of this OTU was indicated in the parentheses. The numbers on the branches are bootstrap values obtained from 1000 bootstrap replicates. AW, sample W; AC, sample C; AL, sample L.

**Figure 6:**
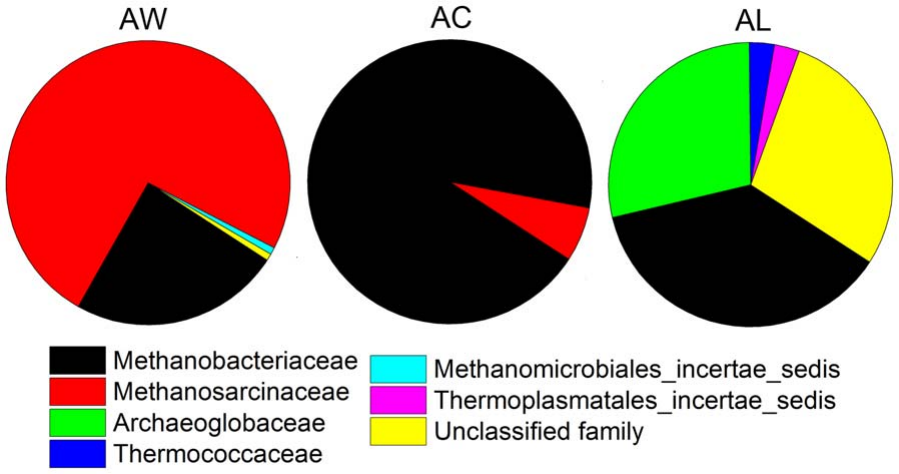
The archaeal composition of injection water and production waters at the family level. AW, sample W; AC, sample C; AL, sample L.

Sequences of three archaeal 16S rRNA gene clone libraries (AC, AL and AW) were analyzed using the same methods used for bacteria. All archaeal sequences belong to Euryarchaeota and were clustered into 4 classes of Methanomicrobia, Methanobacteria, Archaeoglobi and Thermoprotei (Figure 5). In AW, except 2.0% of the sequences were recognized as unclassified families, the remaining sequences were affiliated with Methanosarcinaceae (93.9%) and Methanobacteriaceae (3.1%) (Figure 6). At the genus level, 3.1% of analyzed sequences affiliated with unclassified genus, the remains were affiliated with *Methanomethylovorans* (80.6%), *Methanolobus* (13.3%), *Methanothermobacter* (2.0%) and *Methanobacterium* (1.0%). All of the sequences in AC were affiliated with Methanobacteriaceae (93.8%) and Methanosarcinaceae (6.2%) (Figure 6), of which 93.8% belong to the genus *Methanothermobacter* and 6.2% *Methanomethylovorans*. However, in AL, 28.6% of the sequences were considered unclassified families, while the remains were mainly affiliated with Methanobacteriaceae (37.1%) and Archaeoglobaceae (28.6%) (Figure 6). At the genus level, 34.2% of the total sequences in AL could not be classified to any genus, while the remaining sequences were mainly affiliated to genera *Geoglobus* (28.6%) and *Methanothermobacter* (34.3%).

There were 26 archaeal OTUs total in these three samples (Figure 5 and Figure 4B). Two OTUs (A-OTU3 and A-OTU26) closely related (>98%) to *Methanomethylovorans thermophila* (AY672821), were found in both AW and AC. A-OTU3 had an abundance of 4.0% in AW and 3.1% in AC, and A-OTU26 had an abundance of 57.6% in AW and 3.1% in AC. Other two OTUs (A-OTU16 and A-OTU20), which were related to the classes Methanomicrobia and Methanobacteria respectively, were found in both AW and AL. A-OTU16 had an abundance of 1.0% in AW and 14.3% in AL, and A-OTU20 had an abundance of 1.0% in AW and 5.7% in AL. On the other hand, A-OTU1, related to Methanobacteria, was found in all three samples and had an abundance of 2.0%, 90.6% and 22.9% in AW, AC and AL, respectively. Except for the OTUs described above, 70.6% of OTUs in AW were not detected in either production water. The comparison of archaeal communities showed that A-OTU1 and A-OTU2 were shared by two production waters, of which A-OTU2 presented in production waters only and their abundances in AC and AL were 3.1% and 2.8%, respectively. Additionally, 50% and 83.3% of the total OTUs in AC and AL were unique to themselves, indicating that each production well appears to have a specific archaeal community.

## Discussion

The water flooding method has been widely used to enhance oil recovery, during which the reservoir may be influenced by the microorganisms introduced with the large volumes of injection water [12]. However, little attention has been paid to this issue in water-flooded petroleum reservoirs. In this study, we investigate the microbial communities of injection water and its adjacent production waters in the Gudao petroleum reservoir which has been water flooded for over 30 years.

Our results have indicated that there were a large number of unexplored microorganisms in the subsurface of the Gudao oil field. Clone library analysis showed that 44.9% bacterial sequences and 20% archaeal sequences had less than 97% identity with reference sequences in the database, which seems to be a common phenomenon in petroleum reservoirs. For example, Bonch-Osmolovskaya *et al*. analyzed 15 isolates from the Samotlor oil field and found 4 of them belong to new species [18]. Using the clone library method, Li *et al*. found that 16.7% of bacterial phylotypes and 50% of archaeal phylotypes detected in a high-temperature offshore oil field showed less than 97% sequence similarity with known sequences [19]. These findings indicated the complexity of microbial community of petroleum reservoir and more extensive investigation are still required.

The microbial compositions of injection water and production waters were different according to comparison of the community structure of the three samples. Previously, using PCR-DGGE, She *et al*. found that the similarity of the microbial structure between an injection well and a production well was lower than that of between two production wells [14]; using T-RFLP, Yuan *et al*. indicated that injection water contained more diverse bacteria and archaea compared with production water [15]. In this study, bacterial DGGE fingerprints also showed that the microbial structure of two production waters was more similar when compared to the injection water. However, the detailed information for evaluating the numbers of common microbiota existing in the two types of samples is insufficient when only fingerprinting technology is used. The clone library technology used in this study effectively filled this gap and showed the comprehensive microbial communities in the injection and production wells. The predominant families were distinctly varied between the injection water and the production waters. In more detail, only 7 out of 71 OTUs in the injection water were detected in the production waters indicating that most microorganisms injected into the reservoir were not detected in the microbial communities of production water. On the other hand, the majority of archaeal sequences in the Gudao Oil field were methanogen-related, which is similar to previously reports in other high-temperature petroleum reservoirs [10], [13]. Interestingly, we found that Methanosarcinales, many but not all members of which can use methylamines, H_2_, and acetate, was dominant in the injection water. Methanobacteriales, which prefers H_2_ as substrates for methanogenesis, was predominant in the production waters. These results indicate that both microbial structure and metabolism are different between the injection and production wells.

One reason for the different microbial structures of the injection and production wells might be the distinct difference of temperature, dissolved oxygen, and nutrient availability between the ground water recirculation system and subsurface oil strata [14], [15], [20]. Another reason might be the pore size of the rock matrix. Tiny pore size is restrictive for the transfer of most bacterial cells. Larger microbial cells are more difficultly transported in the formation [21], [22]. There is a sieve effect on microbial cells when injected fluid passes through the subsurface formation [23]. The latter reason is more reasonable for explaining the higher number of injected microbial cells could not be detected among the less dense cells in production water.

Our results also show that a high proportion of unique bacterial and archaeal OTUs exist in the two production waters. The specificities of the two production wells are probably due to their physical and geological properties [12]. The two production wells in the paper were connected to nearby but independent oil-bearing formation layers. Different physiochemical conditions may account for the discrepancy in microbial communities in these oil layers.

Additionally, many sequences obtained from two production wells in this study were affiliated with *Pseudomonas*. The existence of *Pseudomonas* in the thermophilic temperature oil reservoirs were also reported in several previous literatures, such as the Huabei Oil field with in situ temperature 75°C [12], South Elwood field with bottom hole temperature between 70 to 75°C [10], and an offshore petroleum reservoir with temperature 85°C [24]. It is unclear where the sources of these mesophilic microorganisms in the samples are and more knowledge is needed to understand it. Another interesting finding in this study is that the genus *Psychrobacter* existed in the production wells of the Gudao petroleum reservoir. Strains of *Psychrobacter* were previously known to be either psychrophilic, inhabiting in cold environments, such as the Antarctic or the Arctic [25], [26], or mesophilic, isolated from lamb or human lungs [27]. Microorganisms have been repeatedly discovered in environments that do not support their metabolic activity [28], [29]. For example, typical thermophiles were detected in cold environment [30]. However, the function and the existence of the genus *Psychrobacter* in such a high-temperature oil reservoir are still unclear and needs more investigation. The finding that bacteria of this genus degrading long chain n-alkanes [31] raised the possibility of *Psychrobacter* acting as active members in the high temperature petroleum reservoir.

In conclusion, we demonstrated that the microbes in the production water are not significantly related with injected microorganisms and that microbial communities in adjacent production wells were different. These results prompted us to emphasize the importance of monitoring the microorganisms in production water and to understand the effects of the recovery process on the subsurface microbial ecosystem.

## Materials and Methods

### Sample collection and DNA extraction

We obtained permission from the Institute of Oil Recovery Research (Shengli Oil Field Ltd.) for observation and field studies in Gudao Oil Field which has been water flooded since 1974. The re-injection water produced from oil-water separation of production fluids, which collected from production wells in one working block. One water station supplied all the water needed for injection in the working block with several different pump stations to maintain the injection pressure. One injection well (G1–6) and two production wells (3C15 and 6–13) were used in this study. An injection water sample (W) was obtained from the water supply pipelines of G1–6, and production waters (C and L) were oil-water mixtures collected directly from the wellheads of 3C15 and 6–13, respectively. The distances between these three wells ranged from 0.3 to 0.6 km. Both injection water and production waters were full filled in 5 liters sterilized plastic bottles to prevent the diffusion of oxygen into the samples. The plastic bottles were transported to the laboratory at ambient temperature and maintained at 4°C until subsequent analysis.

Demulsification of production waters was completed by adding 1/4 volume of saturated NaCl solution and heating at 70°C for 2 min. The microbial biomass was collected from 250 ml of the oil-water mixture using successive filtration with 8-µm and 0.22-µm filters (Millipore, USA). Genomic DNA was extracted from the filters using a proteinase K/sodium dodecyl sulfate (SDS)/bead beater treatment [32] followed by standard phenol/chloroform extractions [33]. Nucleic acids were purified using the AxyPrep PCR cleanup kit (Axygen Biosciences, USA) and stored at −20°C until 16S rRNA gene amplification.

### Denaturing gradient gel electrophoresis (DGGE) analysis

The bacterial V3 region of the 16S rRNA gene was amplified using the primers P2 and P3 described by Muyzer *et al*. [34]. The 25-µl reaction mixture contained 0.5 U Taq DNA polymerase (Promega, USA), 2.5 µl of the corresponding 10× buffer, 2 µl of a 2.5 mM dNTP mixture (TaKaRa Co., Shiga, Japan), 6.25 pmol of each primer, and 10 ng of genomic DNA. For archaeal V3 region amplification, primers ARCH344f and UNIV522r were used [35], [36]. The 25-µl reaction mixture contained the same components as bacterial PCR. PCR was performed using an initial denaturation at 95°C for 3 min followed by 30 cycles consisting of 95°C for 45 s, 55°C for 30 s, and 72°C for 1 min and a final extension at 72°C for 7 min. PCR amplifications were performed using the Model 475 Gradient Delivery System (Bio-Rad, UK). For both bacterial and archaeal V3 PCR products, “reconditioning PCR” was performed as described by Thompson [37]. The concentrations of the PCR products were determined using a DyNA Quant 200 fluorometer (Pharmacia, US) and evaluated using 1.2% (wt/vol) agarose gel electrophoresis.

A 250-ng aliquot of each V3 PCR product was separated on 8% (wt/vol) denatured polyacrylamide gels using a Dcode System apparatus (Bio-Rad, Hercules, CA); the linear denaturant gradient was 27%–55% for bacteria and 30%–70% for archaea (100% denaturant corresponded to 7 M urea and 40% deionized formamide). Electrophoresis was performed in 1× Tris-acetate-EDTA (TAE) buffer at a constant voltage of 200 V and a temperature of 60°C for 240 min. The DNA bands were stained using SYBR green I (Amresco, Solon, Ohio) and photographed using a UV gel documentation system (UVItec, Cambridge, UK). The PCR-DGGE DNA profiles were digitized, and similarities between samples were analyzed using Quantity One software. Dendrograms were constructed using the unweighted pair group method using arithmetic averages (UPGMA).

### 16S rRNA gene clone library construction

Bacterial and archaeal 16S rRNA gene clone libraries were constructed to examine the microbial communities of injection water and production waters. Universal primers 27F and 1492R [38] were used to amplify bacterial 16S rRNA gene sequences, while primers 46F and 1017R [39] were used to amplify archaeal 16S rRNA gene sequences. The 25-µl PCR reaction mixture contained 0.5 U Taq DNA polymerase (Promega), 2.5 µl of the corresponding 10× buffer, 2 µl of a 2.5 mM dNTP mixture, 1.6 ng/µl BSA, 6.25 pmol of each primer, and 10 ng genomic DNA. All PCR amplifications were performed using the DNA Engine Tetrad 2 Peltier Thermal Cycler (Bio-Rad), and PCR products were evaluated using a 1.2% (wt./vol.) agarose gel. PCR products used for clone library construction were purified using a DNA gel extraction kit (Omega, USA).

The purified PCR products were ligated into a T-vector using the pGEM-T easy vector system I according to the manufacturer's instructions (Promega). The ligation products were transformed into competent *Escherichia coli* DH5α cells (Tiangen, China). Cells were spread on Luria-Bertani agar plates containing 25 µl ampicillin (50 mg/ml), 25 µl X-Gal (0.05 g/ml) and 100 µl IPTG (0.1 M).

### Sequencing analysis of the16S rRNA gene clone libraries

From two clone libraries, 360 bacterial and 166 archaeal white clones were randomly selected for sequencing using an ABI 3730 DNA sequencer. Sequences were assembled after all clones were sequenced in both direction using the primers 27F/1492R for bacteria and 46F/1017R for archaea. Chimeras were detected within the sequence data set using the Chimera Check program (http://greengenes.lbl.gov/cgi-bin/nph-index.cgi). Operational taxonomic units (OTUs) were defined based on 97% similarity using the DOTUR program (http://www.plantpath.wisc.edu/fac/joh/dotur/documentation.html). All sequences were submitted to GenBank for analysis using the BLAST program [40]. Sequences were aligned using Clustal X, version 1.81 [41]. Unifrac was used to determine whether two communities differed significantly using Monte Carlo simulations and measuring the distance between communities [42]. Phylogenetic trees were constructed using the neighbor-joining method [43] within the program Mega 3.1 using 1,000 bootstrap replicates.

Good's formula was used to evaluate whether the libraries constructed were large enough to provide stable phenotype richness as follows:
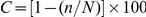
where n is the number of unique clones and N is the total number of clones in each library [44].

To assess the diversity of bacteria and archaea in each sample, two statistical indexes, the Shannon-Wiener index (H) and the Simpson (1-D) index, were calculated using Past [45].

### Nucleotide sequence accession numbers

The GenBank accession numbers for bacterial 16S rRNA gene sequences are FJ900823-FJ901182 and HQ658567-HQ658470; the archaeal 16S rRNA gene sequences are FJ900660-FJ900822 and HQ658471.
